# The chromosome-level genome assembly of lance asiabell (*Codonopsis lanceolata*), a medicinal and vegetable plant of the Campanulaceae family

**DOI:** 10.3389/fgene.2023.1100819

**Published:** 2023-02-01

**Authors:** Woojong Jang, Ji-Nam Kang, Ick-Hyun Jo, Si-Myung Lee, Gyu-Hwang Park, Chang-Kug Kim

**Affiliations:** ^1^ Department of Herbal Crop Research, National Institute of Horticultural and Herbal Science (NIHHS), Rural Development Administration (RDA), Eumseong, South Korea; ^2^ Genomics Division, National Institute of Agricultural Sciences (NAS), Rural Development Administration, Jeonju, South Korea

**Keywords:** Campanulaceae, chromosome-level genome assembly, *Codonopsis lanceolata*, comparative genomics, Hi-C

## Abstract

*Codonopsis lanceolata* (2n = 2x = 16) belongs to the Campanulaceae family and is a valuable medicinal and vegetable plant primarily found in East Asia. Several studies have demonstrated its excellent pharmacological effects, for example in bronchial treatment. However, genomic information of *C. lanceolata* is scarce, hindering studies on crop improvement of the species. Here, we report a high-quality chromosome-level genome assembly of *C. lanceolata* based on a hybrid method using Nanopore long-read, Illumina short-read, and Hi-C data. The assembled genome was completed as 1,273 Mb (84.5% of the estimated genome size), containing eight pseudo-chromosomes, ranging from 101.3 to 184.3 Mb. The genome comprised of 71.3% repeat sequences and 46,005 protein-coding genes, of which 85.7% genes were functionally annotated. Completeness of the assembled genome and genes was assessed to be 97.5% and 90.4%, respectively, by Benchmarking Universal Single-Copy Orthologs analysis. Phylogenetic and synteny analysis revealed that *C. lanceolata* was closely related to *Platycodon grandiflorus* in the Campanulaceae family. Gene family evolution revealed significant expansion of related genes involved in saponin biosynthesis in the *C. lanceolata* genome. This is the first reference genome reported for *C. lanceolata*. The genomic data produced in this study will provide essential information for further research to improve this medicinal plant and will broaden the understanding of the Campanulaceae family.

## 1 Introduction

*Codonopsis lanceolata* (lance asiabell or bonnet bellflower) belongs to the Campanulaceae family which consists of about 2,400 species (Lammers, 2007), and is a perennial vine plant distributed primarily in East Asia. The plant grows up to 1.5 m in moist low mountain or hilly areas (Liu et al., 2019), and has been used as a valuable medicinal and vegetable plant (Lim, 2015). However, climate change and indiscriminate harvesting have resulted in the plant becoming increasingly rare in its natural habitat. This valuable plant exhibits excellent pharmacological properties due to its inherent diverse secondary metabolites such as triterpenoid saponins, phenylpropanoids, alkaloids, polyacetylenes, and other compounds (Hossen et al., 2016; Du et al., 2018). These properties include antioxidant (Jeon et al., 2013), antimicrobial (He et al., 2010), anti-inflammatory (Li et al., 2007), and immune-modulatory (Lee et al., 2007) effects, making the plant highly valuable for commercial use. Moreover, *C. lanceolata* is considered a substitute for *Panax ginseng*, commonly treated as a panacea in Korea.

Several studies have reported the pharmacological effects of *C. lanceolata*, however only a few genetic and genomic studies have been reported. Moreover, limited genomic information on this species is available to guide breeding strategies for crop improvement and to study the conservation of natural populations. The recent development of high-throughput sequencing technologies has reduced the burden on genomic research, making it easily accessible (Pareek et al., 2011; Park and Kim, 2016). The hybrid of Third-Generation Sequencing (TGS) and Next-Generation Sequencing (NGS) technologies such as Oxford Nanopore Technologies (ONT) and short-read sequencing from Illumina have enabled rapid and accurate genome assembly (Lu et al., 2016; Dumschott et al., 2020). These developments provide a suitable opportunity to accumulate genomic information, which is essential for performing various studies related to the minor plants that lack basic research foundations.

Here, we present the first high-quality chromosome-level genome assembly of *C. lanceolata* (2n = 2x = 16) using hybrid methods including NGS, TGS, and high-throughput chromosome conformation capture (Hi-C) technologies. This study provides valuable genomic resources that will enable further research into this medicinal plant and expand our understanding of the Campanulaceae family.

## 2 Materials and methods

### 2.1 Sampling, library construction, and sequencing

Whole plant body of *C. lanceolata* was collected from the National Institute of Horticultural and Herbal Science research field in Eumseong, Korea, and was registered to the National Agrobiodiversity Center (http://genebank.rda.go.kr/) under the voucher number IT239928. The fresh leaves were ground in liquid nitrogen using a mortar and pestle, and genomic DNA was extracted using Exgene Plant SV midi kit (GeneAll Biotechnology, Korea) according to the manufacturer’s instructions. The genomic DNA was purified using ×0.5 AMPure XP bead (Beckman Coulter, United States) according to the manufacturer’s instructions. The quality and quantity of genomic DNA were examined using the Qubit fluorometer (Invitrogen, United States) and Agilent 2200 TapeStation (Agilent Technologies, United States).

An ONT sequencing library was prepared using the ONT 1D ligation sequencing kit SQK-LSK109 (ONT, UK). ONT sequencing was performed using the 1D flowcell vR9.4 and GridION platform operated with MinKNOW software v3.1.20 according to the manufacturer’s instructions. Raw ONT sequencing data (FAST5 files) were converted to FASTQ format using Guppy v2.0.10 (Wick et al., 2019) using default parameters. All Nanopore sequencing procedures were serviced by Phyzen Co. (www.phyzen.com, Korea). An NGS sequencing library was constructed according to the standard Illumina paired-end (PE) library protocol and sequenced using the Illumina HiSeq X platform, all of which were serviced by Macrogen Co. (www.macrogen.com, Korea).

### 2.2 Data trimming and genome size estimation

The ONT sequencing data were trimmed using Porechop v0.2.3 (https://github.com/rrwick/Porechop) using default parameters to remove adaptor and chimeric sequences. Raw Illumina PE data were trimmed using Trimmomatic v0.38 (Bolger et al., 2014) with default parameters. The genome size of *C. lanceolata* was estimated using *k*-mer frequency analyses based on the high-quality Illumina PE data. An optimal *k*-mer value was obtained by Jellyfish v2.0 (Marcais and Kingsford, 2011), and genome size was estimated using GenomeScope v2.0 (Ranallo-Benavidez et al., 2020) based on the 17-mer frequency distribution data.

### 2.3 Genome assembly

The trimmed ONT data were self-corrected using the Canu assembler v1.71 (Koren et al., 2017) with default parameters, and the corrected ONT data were *de novo* assembled using SMARTdenovo (https://github.com/ruanjue/smartdenovo) with a minimum read length of 1,000 bp and other default parameters. The assembled contig sequences were polished twice based on the trimmed PE data using Pilon v1.23 (Walker et al., 2014). An additional polishing process was performed using mapping information of the PE data to improve the assembly quality. The trimmed PE data were mapped to the assembled contig sequences using BWA-MEM v0.7.17 (Li and Durbin, 2009) and Samtools v1.9 (Li et al., 2009) with default parameters. Variant calling was performed using GATK v4.1.4 (https://software.broadinstitute.org/gatk) and Picard v2.20.4 (http://broadinstitute.github.io/picard). Consensus sequence generation through variant substitutions was performed using VCFtools v0.1.13 (https://vcftools.github.io/index.html). Haplotigs in the assembled contig sequences were removed using Purge haplotigs (Roach et al., 2018) with default parameters.

A Hi-C library of *C. lanceolata* was constructed for chromosome-level assembly using Proximo Hi-C Plant Kit (Phase Genomics, United States) according to the manufacturer’s instructions. Crosslinked DNA was digested using the *Sau3A I* restriction enzyme, and proximity ligated with biotinylated nucleotides. The molecules were pulled down with streptavidin beads and processed into an Illumina-compatible sequencing library. Sequencing was performed using the Illumina HiSeq X platform. The generated Hi-C PE data were aligned to the assembled contigs using BWA-MEM v0.7.17 (Li and Durbin, 2009) with -5SP option, and unique mapped reads were detected using SAMBLASTER v0.1.26 (Faust and Hall, 2014) and Samtools v1.9 (Li et al., 2009). A chromosome-level assembly was performed using LACHESIS methods (Burton et al., 2013). All Hi-C assembly procedures were serviced by the Phase Genomics Co. (www.phasegenomics.com, United States). Completeness of the assembled draft genome sequence was validated by NGS data mapping using BWA-MEM v0.7.17 (Li and Durbin, 2009) with default parameters and Benchmarking Universal Single-Copy Orthologs (BUSCO) v5.0.0 (Simao et al., 2015) using the embryophyta_odb10 lineage dataset. LRT Assembly Index (LAI) was also used to assess the genome assembly quality (Qu et al., 2018).

### 2.4 Genome annotation

Initial prediction of repeat sequences in the assembled genome was performed using RepeatModeler v1.0.9 (http://www.repeatmasker.org/RepeatModeler.html), which were merged with previously reported repeat sequences deposited in RepBase v28.04 (https://www.girinst.org/repbase/) to use as a reference repeat database. Consensus repeat sequences in the *C. lanceolata* genome were identified and characterized using RepeatMasker v4.0.9 (http://www.repeatmasker.org) with the custom database.

Gene prediction was carried out based on repeat-masked assembly sequences using an evidence-based annotation method. The protein sequences of four species including *Platycodon grandiflorus* (Jia et al., 2022), *Helianthus annuus* (Badouin et al., 2017), *P. ginseng* (Wang et al., 2022), and *Arabidopsis thaliana* (Cheng et al., 2017) were downloaded from each genome database for homology-based prediction. The transcriptome evidence of two *Codonopsis* species were also collected. The unigene sequences of *C. tangshen* were obtained from a previous study (He et al., 2019). RNA-seq data of *C. pilosula* (Gao et al., 2015) were retrieved from the GenBank Sequence Rad Archive (SRA) database and *de novo* assembled using Trinity v 2.9.1 (Grabherr et al., 2011) with default parameters. Initial gene prediction was performed based on these evidences using MAKER3 v3.01.03 (Holt and Yandell, 2011). The *ab initio* data for final gene prediction was generated using GeneMark-ES v4.38 (Lomsadze et al., 2005), SNAP v2006-07-28 (Zaharia et al., 2011), and AUGUSTUS v3.3.2 (Stanke et al., 2006). The final gene set for *C. lanceolata* was confirmed based on the *ab initio* data using MAKER3 v3.01.03 (Holt and Yandell, 2011) and EvidenceModeler v1.1.1 (Haas et al., 2008).

Functional annotation of the predicted genes was performed by similarities analysis against the NCBI non-redundant (nr) protein database using DIAMOND v0.9.30.131 (Buchfink et al., 2015) with an E-value cutoff of 1e-5. Gene Ontology (GO) terms were assigned to genes using Blast2GO Command Line v1.4.4 (Gotz et al., 2008) with default parameters based on the similarity results. A metabolic pathway was also assigned to genes by searching against the Kyoto Encyclopedia of Genes and Genomes (KEGG) pathway database (Du et al., 2014) using the KEGG Automatic Annotation Server (KAAS) v2.1 (Moriya et al., 2007) with the single-directional best hit (SBH) method and representative gene sets for eukaryotes. Conserved domains within the protein-coding genes were determined using InterProScan v5.34-73.0 (Jones et al., 2014) with default parameters.

### 2.5 Comparative genomic analyses

The collinear blocks within *C. lanceolata* chromosomes and the synteny blocks between *C. lanceolata* and *P. grandiflorus* were identified using MCScanX (Wang et al., 2012) with default parameters. Each block was visualized using Circos (Krzywinski et al., 2009) and SynVisio (https://synvisio.github.io), respectively.

To investigate the phylogenetic status and gene family evolution of *C. lanceolata*, single-copy orthologous genes were searched with eight other species including *Arctium lappa* (Fan et al., 2022), *A. thaliana* (Cheng et al., 2017), *Daucus carota* (Iorizzo et al., 2016), *H. annuus* (Badouin et al., 2017), *Oryza sativa* (Sakai et al., 2013), *P. grandiflorus* (Jia et al., 2022), *Solanum lycopersicum* (Hosmani et al., 2019), and *Vitis vinifera* (Jaillon et al., 2007) using OrthoFinder v2.5.4 (Emms and Kelly, 2019). The divergence time and phylogenetic tree were constructed based on the extracted single-copy orthologous genes among the nine species using BEAST. Analysis of the likelihood for gene family gain and loss of *C. lanceolata* and eight related species was performed using CAFÉ5 (Han et al., 2013).

The total gene set of *C. lanceolata* was compared to those of four other related species including *P. grandiflorus* (Jia et al., 2022), *H. annuus* (Badouin et al., 2017), *P. ginseng* (Wang et al., 2022), and *A. thaliana* (Cheng et al., 2017). Homologous protein sequences were identified using BLASTP analysis with an E-value cutoff of 1e-5. The unique and shared genes among the five species were classified based on the sequence similarity using OrthoVenn2 (Xu et al., 2019) with plants group parameters. GO enrichment analysis was performed on the shared genes among the five species and the unique genes in *C. lanceolata*. Candidate genes encoding enzymes involved in saponin biosynthesis were identified by searching genes assigned to the sesquiterpenoid and triterpenoid biosynthesis pathway (https://www.genome.jp/dbget-bin/www_bget?ko00909) using KAAS analysis. Phylogenetic analysis based on the candidate genes was performed using the maximum likelihood (ML) method with 1,000 bootstraps using MEGA v11 (Tamura et al., 2021) with default parameters after aligning predicted amino acid sequences using MUSCLE (Edgar, 2004) with default parameters.

## 3 Results and discussion

**FIGURE 1:**
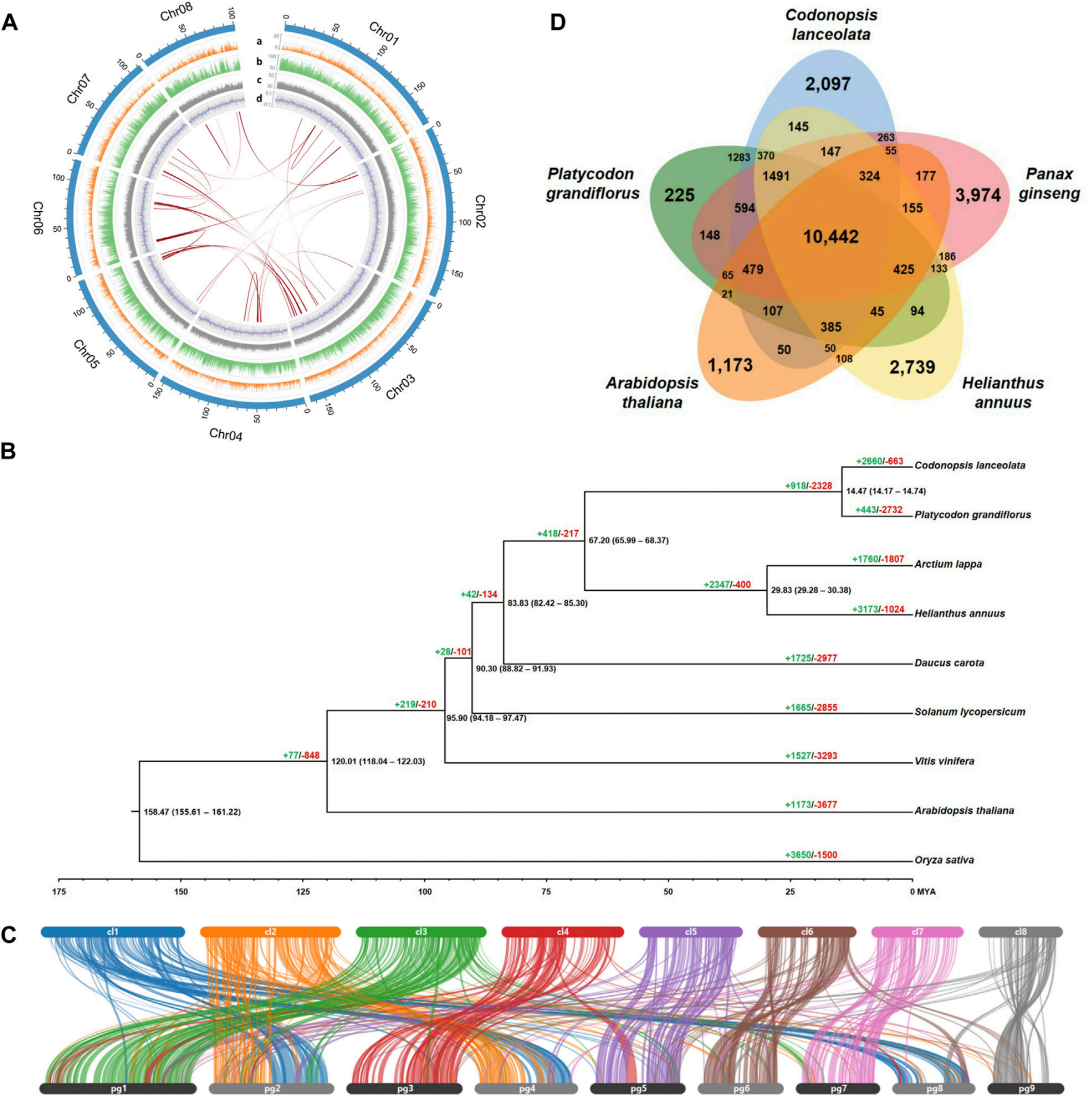
*C. lanceolata* genome characteristics, phylogenomics, synteny structure, and gene clusters. **(A)** Circos plot representing the eight pseudo-chromosomes in the *C. lanceolata* assembly and their features. The outermost track (light blue) indicates eight pseudo-chromosomes. Tracks a–d depict the distribution of genes (orange), repeat elements (green), GC contents (gray), and GC skew (blue), respectively. The innermost lines (red) represent collinear blocks identified within the *C. lanceolata* genome. **(B)** Phylogenetic tree and gene family evolution of nine plant species. The black numbers above each node indicate divergence times between groups. The green (+) and red (−) numbers indicate the values of expanded and contracted gene families, respectively. **(C)** Synteny blocks between *C. lanceolata* and *P. grandiflorus* genomes. The upper and lower bars indicate each pseudo-chromosomes of *C. lanceolata* and *P. grandifloras*, respectively. **(D)** Shared and unique gene clusters in *C. lanceolata* and four related species.

Approximately 61.2 Gb Nanopore long-reads with an average read length of 4,423 bp and 104.9 Gb Illumina short-reads were generated after the trimming process from raw sequencing data for genome assembly of *C. lanceolata* (Supplementary Table S1). The *C. lanceolata* genome was estimated to be about 1,507 Mb, with 1.74% heterozygosity and 82.03% repeat sequences, based on optimal 17-mer analysis using high-quality Illumina short-reads (Supplementary Figure S1; Supplementary Table S2). Initial draft sequences of 1,272 Mb, consisting of 19,667 contigs showing N50 value of 88.7 kb (Supplementary Table S3), were assembled based on Nanopore long-reads used as the seed sequences through a polishing process using Illumina short-reads. Finally, a chromosome-level genome assembly for *C. lanceolata*, that was 1,273 Mb (84.5% of estimated genome size) and composed of 4,828 scaffolds with N50 value of 154.4 Mb, was completed through a scaffolding process using 47.1 Gb Illumina data produced from Hi-C library (Table 1; Supplementary Table S1). The longest eight scaffolds, ranging in length from 101.3 to 184.3 Mb, included 90.1% (1,147 Mb) of the completed assembled genome sequence (Figure 1A; Supplementary Table S4). The Hi-C interaction heatmap showed distinct interaction signals that distinguished eight pseudo-chromosomes within each pseudo-chromosome (Supplementary Figure S2). BUSCO analysis assessed that the assembled draft genome sequence captured 1,574 (97.5%) complete BUSCOs including 1,476 (91.4%) single-copy BUSCOs, 98 (6.1%) duplicated BUSCOs, and 21 (1.3%) fragmented BUSCOs (Table 1). LAI for assembly quality assessment of repetitive sequences in the draft genome sequence was calculated as 9.08. These results demonstrated that the *C. lanceolata* genome sequence completed in this study was assembled with a high-quality of completeness.

**TABLE 1:** Summary statistics of genome assembly and gene prediction of *C. lanceolata*.

Genome assembly	
Total Genome Length	1,273,258,064
Scaffold No.	4,828
Scaffold N50 (bp)	154,401,475
Complete BUSCOs (%)	97.5
Complete and Single-copy BUSCOs (%)	91.4
Complete and Duplicated BUSCOs (%)	6.1
Fragmented BUSCOs (%)	1.3
Missing BUSCOs (%)	1.2
Gene Prediction	
Protein-Coding Gene No.	46,005
Total Gene Length (bp)	42,414,642
Average Gene Length (bp)	3,568
Average Exon Length (bp)	922
Average Intron Length (bp)	2,646
GC Content (%)	44.04

The genome annotation characterized 908.3 Mb repeat sequences in the *C. lanceolata* genome, accounting for 71.3% of the genome (Supplementary Table S5). Among the various repeat elements, long terminal repeats (LTRs), especially *Gypsy* (17.0%) and *Copia* (11.5%) type, were remarkably prevalent in the genome. A total of 46,005 genes were predicted based on protein and transcriptome evidence in the *C. lanceolata* genome (Table 1). The total length of the gene set was 42.41 Mb with an average length of 3,568 bp, and GC content of 44.04%. Average exon and intron length of the gene set were calculated as 922 bp and 2,646 bp, respectively. Among them, 39,435 genes (85.7%) could be functionally annotated by comparing their homology against libraries of known proteins (Supplementary Table S6).

In order to detect the degree of duplication, collinear blocks within the *C. lanceolata* chromosomes were searched using the annotated gene information. A total of 27 collinear blocks were identified, indicating that there were few duplication events in the entire *C. lanceolata* genome (Figure 1A; Supplementary Table S7).

Phylogenetic analysis based on 844 single-copy orthologous genes showed that *C. lanceolata* was closely related to *P. grandiflorus* in the Campanulaceae family (Figure 1B). The 14.47 MYA of divergence time between two species corresponded with the synteny result indicating that the gene structures and contents were highly conserved each other (Figures 1B, C). Gene family evolution among the nine species by CAFÉ analysis revealed that 2660 and 663 gene families were significantly expanded and contracted in the *C. lanceolata* genome, respectively.

Gene clustering analysis based on similarity among protein sequences revealed that the *C. lanceolata* gene products were grouped into 10,442 gene clusters with shared genes from *P. grandiflorus*, *P. ginseng*, *H. annuus*, and *A. thaliana*, as well as 2,097 clusters with genes unique to *C. lanceolata* (Figure 1D). GO enrichment analysis of the shared clusters identified the abundant GO terms, such as the biological process GO terms related to regulation of transcription, RNA modification, and rRNA processing, as well as molecular function GO terms related to oxidoreductase activity, oxidoreductase activity, and carboxylic ester hydrolase activity (Supplementary Table S8). In the clusters with genes unique to *C. lanceolata*, biological process GO terms related to terpenoid biosynthetic process were abundant (Supplementary Table S9). A total of 106 candidate genes involved in the saponin biosynthesis pathway were identified using KAAS analysis (Supplementary Table S10). Of these, putative beta-amyrin synthase genes that are important oxidosqualene cyclases for triterpenoid saponin biosynthesis were identified to be expanded and distinctly grouped in *C. lanceolata* compared to the other four plant species examined (Supplementary Figure S3).

## Data Availability

The datasets presented in this study can be found in online repositories. The names of the repository/repositories and accession number(s) can be found below: https://www.ncbi.nlm.nih.gov/, PRJNA627046; https://figshare.com/, 10.6084/m9.figshare.21507774.
