# Supplementary material for: The chromosome-level genome assembly of lance asiabell (Codonopsis lanceolata), a medicinal and vegetable plant of the Campanulaceae family
Source: Front Genet. 2023 Feb 1;14:1100819. doi: 10.3389/fgene.2023.1100819 (PMC9929348; doi:10.3389/fgene.2023.1100819)
Supplement: Supplementary file 1 [file DataSheet1.docx]

***Supplementary Material***

# Supplementary Figures and Tables

## Supplementary Figures

**
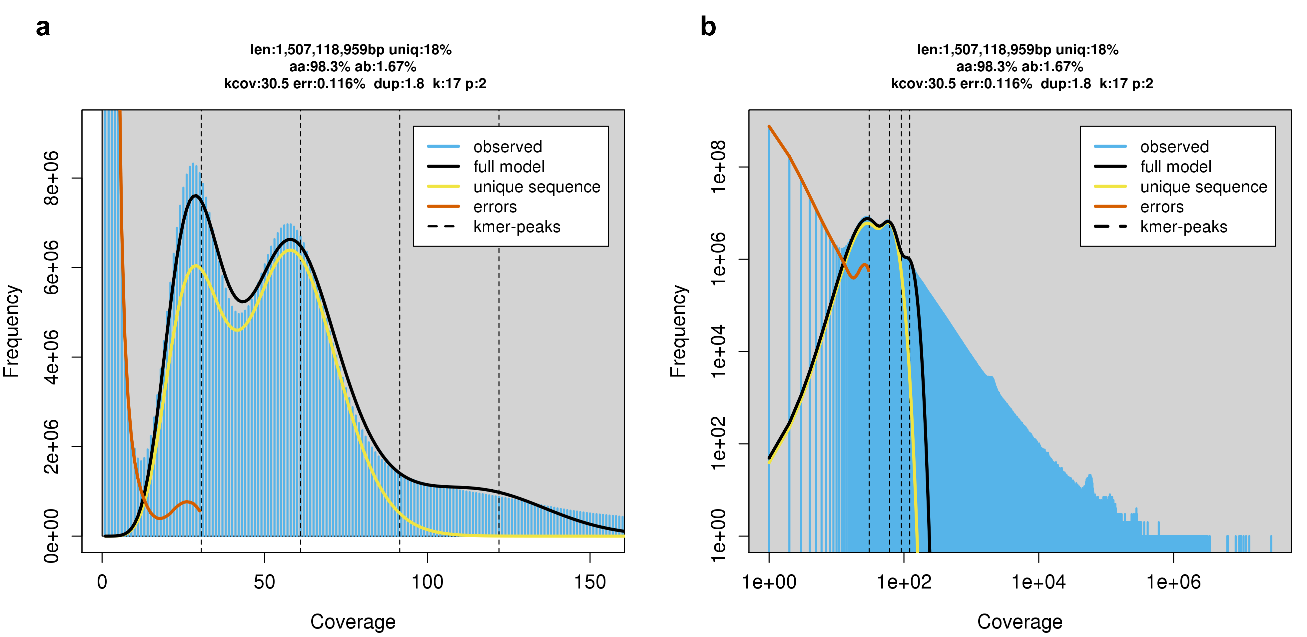
**

**Supplementary Figure S1**. GenomeScope profile plots of *k*-mer frequency. (a) *k*-mer frequency and (b) log-transformed *k*-mer coverage at a *k*-mer length of 17.


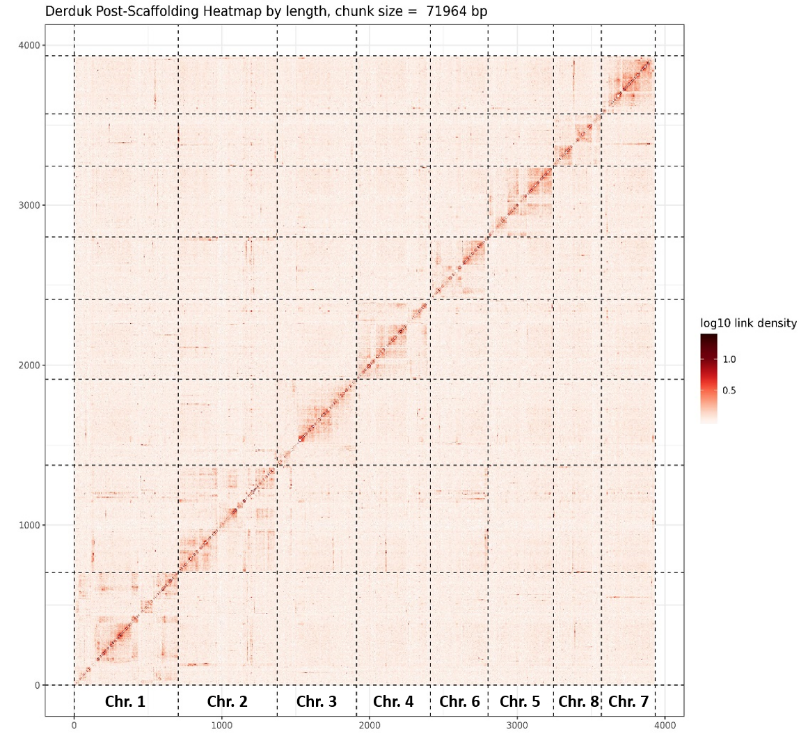


**Supplementary Figure S2**. Hi-C interaction heatmap for the *C. lanceolata* genome.


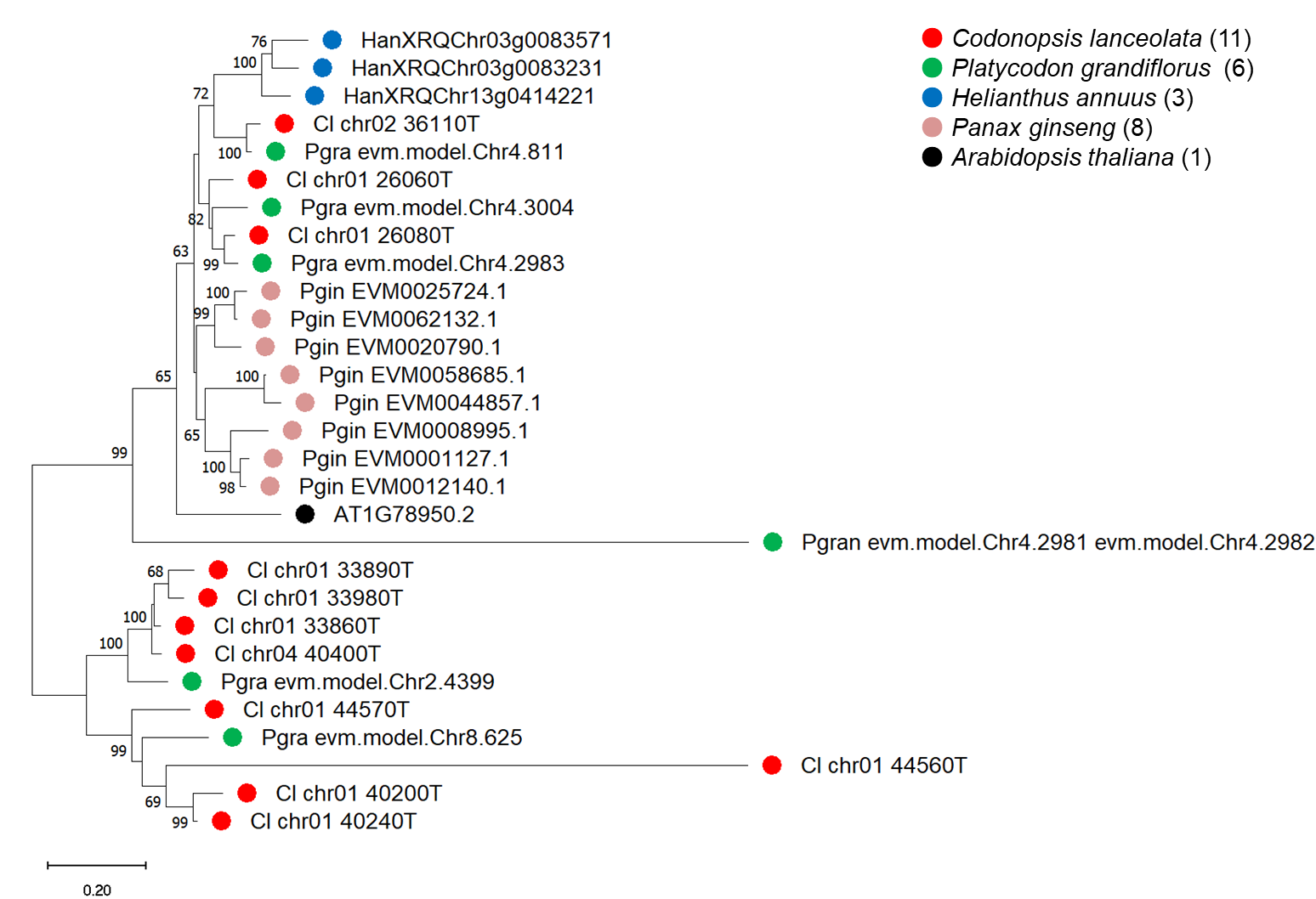


**Supplementary Figure S3**. Phylogenetic tree of *beta*-amyrin synthases identified in *C. lanceolata* and four related species. The number of genes was indicated in parentheses after each species names.

## Supplementary Tables

**Supplementary Table S1**. Statistics summary of sequencing data used for *C. lanceolata* genome assembly.

| **Sequencing Technologies and Platforms** | **Raw data** | | | **Trimmed Data** | | | | **Corrected data (using Canu)** | | | |
| --- | --- | --- | --- | --- | --- | --- | --- | --- | --- | --- | --- |
|  | **Total Amount (Gb)** | **Total reads** | **Average Read Length (bp)** | **Total Amount (Gb)** | **Total reads** | **Average Read Length (bp)** | **Coverage* (X)** | **Total Amount (Gb)** | **Total reads** | **Average Read Length (bp)** | **Coverage* (X)** |
| TGS  ONT GridION | 61.7 | 13,872,458 | 4,451 | 61.2 | 13,844,353 | 4,423 | 40.6 | 48.6 | 9,103,986 | 5,341 | 32.2 |
| NGS  Illumina HiSeq X | 111.9 | 741,345,074 | 151 | 104.9 | 732,886,858 | 143 | 69.6 | - | - | - | - |
| Hi-C | 47.1 | 314,309,040 | 151 | - | - | - | - | - | - | - | - |

*Coverage values was estimated based on the estimated genome size by *k*-mer analysis.

NGS, Next-Generation Sequencing; ONT, Oxford Nanopore Technologies; TGS, Third-Generation Sequencing

**Supplementary Table S2**. Genome size estimation and characteristics calculated by *k*-mer frequency analysis.

| **K-mer** | **Genome Size (Mb)** | **Heterozygous Rate (%)** | **Repeat Rate (Mb / %)** |
| --- | --- | --- | --- |
| 17 | 1,507 | 1.74 | 1,236 / 82.03 |

**Supplementary Table S3**. Summary statistics of initial draft sequence before scaffolding process.

| **Contig Number** | 19,667 |
| --- | --- |
| **Total Sequence Length (bp)** | 1,271,819,386 |
| **Min Length (bp)** | 4,544 |
| **Max Length (bp)** | 947,918 |
| **Average Length (bp)** | 64,668 |
| **N50 (bp)** | 88,675 |
| **N90 (bp)** | 30,248 |
| **Complete BUSCOs (%)** | 97.2 |

BUSCO, Benchmarking Universal Single-Copy Orthologs

**Supplementary Table S4**. Length and merged contigs information for the eight longest scaffolds corresponding to pseudochromosomes.

| **Chromosome No.** | **Assigned Contigs Number** | **Length (bp)** |
| --- | --- | --- |
| Chr. 1 | 2,307 | 184,336,258 |
| Chr. 2 | 2,160 | 179,644,557 |
| Chr. 3 | 2,057 | 165,490,992 |
| Chr. 4 | 2,119 | 154,401,575 |
| Chr. 5 | 1,768 | 128,354,623 |
| Chr. 6 | 1,562 | 121,454,794 |
| Chr. 7 | 1,555 | 112,340,686 |
| Chr. 8 | 1,317 | 101,348,483 |
| **Total** | **14,845** | **1,147,371,968** |

**Supplementary Table S5**. Statistics summary of repeat annotation.

| **Type** | **Count** | **Total Length (bp)** | **Coverage* (X)** |
| --- | --- | --- | --- |
| DNA Elements | 326,301 | 90,731,737 | 0.071 |
| LINEs | 71,581 | 54,011,533 | 0.042 |
| SINEs | 20,142 | 2,736,750 | 0.002 |
| LTR/Gypsy | 206,014 | 216,554,301 | 0.170 |
| LTR/Copia | 133,960 | 146,629,886 | 0.115 |
| LTR/Others | 129,693 | 51,805,567 | 0.041 |
| Simple Repeat | 186,706 | 18,948,496 | 0.015 |
| Others | 59,826 | 13,432,820 | 0.011 |
| Unknown | 1024,335 | 313,490,248 | 0.246 |
| **Total** | **2,158,558** | **908,341,338** | **0.713** |

*Coverage values was estimated based on the assembled genome length.

LINE, Long Interspersed Element; LTR, Long Terminal Repeat; SINE, Short Interspersed Element

**Supplementary Table S6**. Statistics summary of functional annotation.

| **Databases** | **Program and Parameters** | **Annotated Genes Number** | **Coverage* (%)** |
| --- | --- | --- | --- |
| NCBI nr proteins | DIAMOND, E-value cutoff 1e-5 | 39,194 | 85.20 |
| Protein Domains | InterProScan, default parameters | 29,661 | 64.47 |
| Gene Ontology (GO) | Blast2GO, default parameters | 21,998 | 47.82 |
| KEGG Pathway | KAAS, SBH method | 14,982 | 32.57 |
| **Total** | - | **39,435** | **85.72** |

* Coverage values was estimated based on the predicted genes number in the assembled genome.

KAAS, KEGG Automatic Annotation Server; KEGG, Kyoto Encyclopedia of Genes and Genomes; nr, non-redundant; SBH, Single-directional Best Hit

**Supplementary Table S7**. Summary information of collinear block in the *C. lanceolata* genome.

| **Block No.** | **Synteny Block A** | | | | **Synteny Block B** | | | |
| --- | --- | --- | --- | --- | --- | --- | --- | --- |
|  | **Chr.** | **Start (bp)** | **End (bp)**  **L** | **Length (bp)** | **Chr.** | **Start (bp)** | **End (bp)**  **L** | **Length (bp)** |
| 1 | 1 | 82,557,640 | 83,767,113 | 1,209,473 | 1 | 98,368,289 | 99,133,221 | 764,932 |
| 2 | 1 | 54,884,255 | 55,569,046 | 684,791 | 3 | 63,251,145 | 63,580,838 | 329,693 |
| 3 | 1 | 127,610,255 | 128,138,985 | 528,730 | 4 | 49,839,908 | 50,642,277 | 802,369 |
| 4 | 1 | 143,451,811 | 144,805,651 | 1,353,840 | 5 | 5,913,237 | 6,405,246 | 492,009 |
| 5 | 1 | 82,338,384 | 83,283,369 | 944,985 | 7 | 52,667,990 | 53,286,669 | 618,679 |
| 6 | 2 | 50,830,799 | 51,330,297 | 499,498 | 3 | 106,050,588 | 106,998,531 | 947,943 |
| 7 | 2 | 46,017,740 | 46,355,695 | 337,955 | 5 | 12,948,180 | 13,551,604 | 603,424 |
| 8 | 2 | 8,812,367 | 9,730,849 | 918,482 | 6 | 16,570,617 | 18,118,826 | 1,548,209 |
| 9 | 2 | 15,126,130 | 16,788,566 | 1,662,436 | 8 | 88,159,556 | 89,563,335 | 1,403,779 |
| 10 | 3 | 102,464,895 | 103,222,814 | 757,919 | 4 | 43,717,493 | 44,173,545 | 456,052 |
| 11 | 3 | 7,104,172 | 7,811,527 | 707,355 | 4 | 69,331,053 | 69,883,646 | 552,593 |
| 12 | 3 | 147,341,637 | 148,696,483 | 1,354,846 | 5 | 114,547,365 | 115,436,127 | 888,762 |
| 13 | 3 | 137,401,568 | 140,001,192 | 2,599,624 | 5 | 116,370,705 | 118,802,357 | 2,431,652 |
| 14 | 3 | 127,125,575 | 128,807,475 | 1,681,900 | 6 | 86,151,830 | 88,652,403 | 2,500,573 |
| 15 | 3 | 86,374,972 | 86,908,962 | 533,990 | 6 | 84,292,157 | 85,096,529 | 804,372 |
| 16 | 3 | 151,365,804 | 152,954,690 | 1,588,886 | 7 | 20,245,504 | 22,426,832 | 2,181,328 |
| 17 | 3 | 157,840,910 | 158,574,595 | 733,685 | 7 | 13,938,195 | 14,647,107 | 708,912 |
| 18 | 4 | 50,743,331 | 52,540,930 | 1,797,599 | 4 | 75,762,966 | 76,925,224 | 1,162,258 |
| 19 | 4 | 44,859,874 | 46,210,687 | 1,350,813 | 5 | 122,754,954 | 123,881,747 | 1,126,793 |
| 20 | 5 | 112,769,432 | 113,665,973 | 896,541 | 6 | 84,448,282 | 85,451,886 | 1,003,604 |
| 21 | 5 | 112,306,672 | 113,008,378 | 701,706 | 6 | 84,173,461 | 84,759,646 | 586,185 |
| 22 | 6 | 13,828,758 | 14,895,807 | 1,067,049 | 6 | 15,002,690 | 17,198,050 | 2,195,360 |
| 23 | 6 | 76,067,213 | 77,081,904 | 1,014,691 | 7 | 69,591,309 | 70,547,545 | 956,236 |
| 24 | 6 | 105,464,368 | 105,907,319 | 442,951 | 7 | 41,258,083 | 41,864,877 | 606,794 |
| 25 | 6 | 105,480,325 | 105,931,618 | 451,293 | 8 | 83,213,407 | 83,757,741 | 544,334 |
| 26 | 7 | 38,123,340 | 38,269,870 | 146,530 | 8 | 43,376,144 | 43,561,385 | 185,241 |
| 27 | 8 | 21,014,654 | 21,807,928 | 793,274 | 8 | 22,000,629 | 22,666,942 | 666,313 |

Chr., Chromosome

**Supplementary Table S8**. GO enrichment analysis of shared genes among *C. lanceolata* and four related species using OrthoVenn2.

| **GO ID** | **GO Term** | **GO Class** | **Count** | **Adjusted**  **P-Value** |
| --- | --- | --- | --- | --- |
| GO:0006355 | Regulation of transcription, DNA-templated | BP | 199 | 0.000623497 |
| GO:0009451 | RNA modification | BP | 95 | 3.61E-09 |
| GO:0006364 | rRNA processing | BP | 75 | 9.52E-11 |
| GO:0006486 | Protein glycosylation | BP | 65 | 0.000599335 |
| GO:0006952 | Defense response | BP | 52 | 5.18E-10 |
| GO:0006397 | mRNA processing | BP | 44 | 2.45633E-05 |
| GO:0046777 | Protein autophosphorylation | BP | 41 | 0.000569637 |
| GO:0015979 | Photosynthesis | BP | 35 | 0.000467064 |
| GO:0000398 | mRNA splicing, via spliceosome | BP | 32 | 0.00020662 |
| GO:0007165 | Signal transduction | BP | 31 | 0.00013281 |
| GO:0010027 | Thylakoid membrane organization | BP | 23 | 7.13046E-05 |
| GO:0010200 | Response to chitin | BP | 16 | 6.6007E-05 |
| GO:0045944 | Positive regulation of transcription | BP | 15 | 6.50388E-05 |
| GO:0097428 | Protein maturation by iron-sulfur cluster transfer | BP | 9 | 0.000546014 |
| GO:0048544 | Recognition of pollen | BP | 8 | 8.31E-09 |
| GO:0009734 | Auxin-activated signaling pathway | BP | 8 | 0.000401365 |
| GO:0009607 | Response to biotic stimulus | BP | 7 | 3.48108E-05 |
| GO:0009626 | Plant-type hypersensitive response | BP | 4 | 0.000359946 |
| GO:0050832 | Defense response to fungus | BP | 3 | 3.98417E-06 |
| GO:0015074 | DNA integration | BP | 2 | 6.45E-15 |
| GO:0031640 | Killing of cells of other organism | BP | 2 | 1.44E-12 |
| GO:0009820 | Alkaloid metabolic process | BP | 2 | 7.64E-07 |
| GO:0009791 | Post-embryonic development | BP | 2 | 7.06932E-05 |
| GO:0071230 | Cellular response to amino acid stimulus | BP | 2 | 0.000115401 |
| GO:0016021 | Integral component of membrane | CC | 89 | 0.000800357 |
| GO:0016491 | Oxidoreductase activity | MF | 32 | 2.43E-07 |
| GO:0003677 | DNA binding | MF | 23 | 0.000735076 |
| GO:0016705 | Oxidoreductase activity, acting on paired donors | MF | 9 | 1.42E-19 |
| GO:0052689 | Carboxylic ester hydrolase activity | MF | 6 | 0.000493923 |
| GO:0045735 | Nutrient reservoir activity | MF | 4 | 2.53E-05 |
| GO:0016709 | Oxidoreductase activity, acting on paired donors | MF | 3 | 0.000679822 |

BP, Biological Process; CC, Cellular Component; GO, Gene Ontology; MF, Molecular Function

**Supplementary Table S9**. GO enrichment analysis of unique genes in *C. lanceolata* using OrthoVenn2.

| **GO ID** | **GO Term** | **GO Class** | **Count** | **Adjusted**  **P-Value** |
| --- | --- | --- | --- | --- |
| GO:0046777 | Protein autophosphorylation | BP | 18 | 1.00E-04 |
| GO:0032196 | Transposition | BP | 8 | 6.07E-07 |
| GO:0016114 | Terpenoid biosynthetic process | BP | 6 | 2.21E-04 |
| GO:0043693 | Monoterpene biosynthetic process | BP | 4 | 3.61E-04 |
| GO:1902242 | Copal-8-ol diphosphate(3-) catabolic process | BP | 3 | 1.26E-04 |
| GO:0016705 | Oxidoreductase activity, acting on paired donors, with incorporation or reduction of molecular oxygen | MF | 19 | 1.11E-05 |
| GO:0016709 | Oxidoreductase activity, acting on paired donors, with incorporation or reduction of molecular oxygen, NAD(P)H as one donor, and incorporation of one atom of oxygen | MF | 7 | 1.52E-04 |

BP, Biological Process; GO, Gene Ontology; MF, Molecular Function

**Supplementary Table S10**. Genes assigned to the sesquiterpenoid and triterpenoid biosynthesis pathway in *C. lanceolata* genome.

| **Gene ID** | **Protein Length (aa)** | **KO** | **KO Description** |
| --- | --- | --- | --- |
| Cl_C14884_unscaffolded_00030T | 496 | K15803 | GERD; (-)-germacrene D synthase [EC:4.2.3.75] |
| Cl_C16644_unscaffolded_00010T | 140 | K15803 | GERD; (-)-germacrene D synthase [EC:4.2.3.75] |
| Cl_C16644_unscaffolded_00020T | 233 | K15803 | GERD; (-)-germacrene D synthase [EC:4.2.3.75] |
| Cl_C18016_unscaffolded_00010T | 625 | K20659 | LUS; lupeol synthase [EC:5.4.99.41] |
| Cl_C18938_unscaffolded_00010T | 89 | K14184 | TPS21; alpha-humulene/beta-caryophyllene synthase [EC:4.2.3.104 4.2.3.57] |
| Cl_C19165_unscaffolded_00050T | 182 | K20659 | LUS; lupeol synthase [EC:5.4.99.41] |
| Cl_C20277_unscaffolded_00010T | 578 | K20659 | LUS; lupeol synthase [EC:5.4.99.41] |
| Cl_C20733_unscaffolded_00010T | 458 | K20659 | LUS; lupeol synthase [EC:5.4.99.41] |
| Cl_C21315_unscaffolded_00010T | 269 | K15803 | GERD; (-)-germacrene D synthase [EC:4.2.3.75] |
| Cl_chr01_03360T | 128 | K15813 | LUP4; beta-amyrin synthase [EC:5.4.99.39] |
| Cl_chr01_26050T | 183 | K15813 | LUP4; beta-amyrin synthase [EC:5.4.99.39] |
| Cl_chr01_26060T | 549 | K15813 | LUP4; beta-amyrin synthase [EC:5.4.99.39] |
| Cl_chr01_26080T | 763 | K15813 | LUP4; beta-amyrin synthase [EC:5.4.99.39] |
| Cl_chr01_33860T | 767 | K15813 | LUP4; beta-amyrin synthase [EC:5.4.99.39] |
| Cl_chr01_33890T | 504 | K15813 | LUP4; beta-amyrin synthase [EC:5.4.99.39] |
| Cl_chr01_33970T | 126 | K15822 | CAMS1, LUP3; camelliol C synthase [EC:5.4.99.38] |
| Cl_chr01_33980T | 597 | K15813 | LUP4; beta-amyrin synthase [EC:5.4.99.39] |
| Cl_chr01_36580T | 165 | K15803 | GERD; (-)-germacrene D synthase [EC:4.2.3.75] |
| Cl_chr01_36590T | 132 | K14184 | TPS21; alpha-humulene/beta-caryophyllene synthase [EC:4.2.3.104 4.2.3.57] |
| Cl_chr01_37390T | 340 | K15800 | GAO; germacrene A oxidase [EC:1.14.14.95] |
| Cl_chr01_38840T | 158 | K15800 | GAO; germacrene A oxidase [EC:1.14.14.95] |
| Cl_chr01_38850T | 209 | K15800 | GAO; germacrene A oxidase [EC:1.14.14.95] |
| Cl_chr01_40200T | 514 | K15813 | LUP4; beta-amyrin synthase [EC:5.4.99.39] |
| Cl_chr01_40240T | 763 | K15813 | LUP4; beta-amyrin synthase [EC:5.4.99.39] |
| Cl_chr01_44560T | 308 | K15813 | LUP4; beta-amyrin synthase [EC:5.4.99.39] |
| Cl_chr01_44570T | 419 | K15813 | LUP4; beta-amyrin synthase [EC:5.4.99.39] |
| Cl_chr01_46720T | 112 | K20659 | LUS; lupeol synthase [EC:5.4.99.41] |
| Cl_chr01_56720T | 510 | K15800 | GAO; germacrene A oxidase [EC:1.14.14.95] |
| Cl_chr01_60880T | 84 | K14184 | TPS21; alpha-humulene/beta-caryophyllene synthase [EC:4.2.3.104 4.2.3.57] |
| Cl_chr02_19690T | 412 | K00511 | SQLE, ERG1; squalene monooxygenase [EC:1.14.14.17] |
| Cl_chr02_19700T | 134 | K00511 | SQLE, ERG1; squalene monooxygenase [EC:1.14.14.17] |
| Cl_chr02_36110T | 763 | K15813 | LUP4; beta-amyrin synthase [EC:5.4.99.39] |
| Cl_chr02_41830T | 245 | K14173 | AFS1; alpha-farnesene synthase [EC:4.2.3.46] |
| Cl_chr02_49740T | 338 | K15891 | FLDH; NAD+-dependent farnesol dehydrogenase [EC:1.1.1.354] |
| Cl_chr02_56040T | 60 | K15800 | GAO; germacrene A oxidase [EC:1.14.14.95] |
| Cl_chr02_63480T | 342 | K15800 | GAO; germacrene A oxidase [EC:1.14.14.95] |
| Cl_chr03_01200T | 562 | K14173 | AFS1; alpha-farnesene synthase [EC:4.2.3.46] |
| Cl_chr03_01240T | 304 | K14175 | NES1; (3S,6E)-nerolidol synthase [EC:4.2.3.48] |
| Cl_chr03_01250T | 233 | K14175 | NES1; (3S,6E)-nerolidol synthase [EC:4.2.3.48] |
| Cl_chr03_01280T | 536 | K14175 | NES1; (3S,6E)-nerolidol synthase [EC:4.2.3.48] |
| Cl_chr03_07740T | 78 | K15822 | CAMS1, LUP3; camelliol C synthase [EC:5.4.99.38] |
| Cl_chr03_15570T | 563 | K14173 | AFS1; alpha-farnesene synthase [EC:4.2.3.46] |
| Cl_chr03_23720T | 312 | K00511 | SQLE, ERG1; squalene monooxygenase [EC:1.14.14.17] |
| Cl_chr03_23730T | 262 | K00511 | SQLE, ERG1; squalene monooxygenase [EC:1.14.14.17] |
| Cl_chr03_27130T | 533 | K14175 | NES1; (3S,6E)-nerolidol synthase [EC:4.2.3.48] |
| Cl_chr03_27140T | 377 | K14173 | AFS1; alpha-farnesene synthase [EC:4.2.3.46] |
| Cl_chr03_27150T | 527 | K14175 | NES1; (3S,6E)-nerolidol synthase [EC:4.2.3.48] |
| Cl_chr03_27170T | 94 | K14173 | AFS1; alpha-farnesene synthase [EC:4.2.3.46] |
| Cl_chr03_27180T | 566 | K14175 | NES1; (3S,6E)-nerolidol synthase [EC:4.2.3.48] |
| Cl_chr03_27200T | 361 | K14175 | NES1; (3S,6E)-nerolidol synthase [EC:4.2.3.48] |
| Cl_chr03_27220T | 491 | K14173 | AFS1; alpha-farnesene synthase [EC:4.2.3.46] |
| Cl_chr03_27360T | 451 | K14175 | NES1; (3S,6E)-nerolidol synthase [EC:4.2.3.48] |
| Cl_chr04_00090T | 537 | K00511 | SQLE, ERG1; squalene monooxygenase [EC:1.14.14.17] |
| Cl_chr04_00100T | 526 | K00511 | SQLE, ERG1; squalene monooxygenase [EC:1.14.14.17] |
| Cl_chr04_00920T | 550 | K20659 | LUS; lupeol synthase [EC:5.4.99.41] |
| Cl_chr04_07000T | 141 | K00801 | FDFT1; farnesyl-diphosphate farnesyltransferase [EC:2.5.1.21] |
| Cl_chr04_07960T | 73 | K20659 | LUS; lupeol synthase [EC:5.4.99.41] |
| Cl_chr04_07970T | 73 | K20659 | LUS; lupeol synthase [EC:5.4.99.41] |
| Cl_chr04_11290T | 674 | K20659 | LUS; lupeol synthase [EC:5.4.99.41] |
| Cl_chr04_33120T | 81 | K20659 | LUS; lupeol synthase [EC:5.4.99.41] |
| Cl_chr04_40400T | 708 | K15813 | LUP4; beta-amyrin synthase [EC:5.4.99.39] |
| Cl_chr04_40910T | 424 | K15803 | GERD; (-)-germacrene D synthase [EC:4.2.3.75] |
| Cl_chr05_13260T | 81 | K20659 | LUS; lupeol synthase [EC:5.4.99.41] |
| Cl_chr05_13270T | 90 | K20659 | LUS; lupeol synthase [EC:5.4.99.41] |
| Cl_chr05_13990T | 424 | K00801 | FDFT1; farnesyl-diphosphate farnesyltransferase [EC:2.5.1.21] |
| Cl_chr05_14040T | 418 | K00801 | FDFT1; farnesyl-diphosphate farnesyltransferase [EC:2.5.1.21] |
| Cl_chr05_22180T | 95 | K20659 | LUS; lupeol synthase [EC:5.4.99.41] |
| Cl_chr05_23220T | 114 | K20659 | LUS; lupeol synthase [EC:5.4.99.41] |
| Cl_chr05_28360T | 550 | K00511 | SQLE, ERG1; squalene monooxygenase [EC:1.14.14.17] |
| Cl_chr05_41810T | 155 | K20659 | LUS; lupeol synthase [EC:5.4.99.41] |
| Cl_chr06_07170T | 644 | K14175 | NES1; (3S,6E)-nerolidol synthase [EC:4.2.3.48] |
| Cl_chr06_19010T | 347 | K20659 | LUS; lupeol synthase [EC:5.4.99.41] |
| Cl_chr06_21580T | 248 | K15803 | GERD; (-)-germacrene D synthase [EC:4.2.3.75] |
| Cl_chr06_21590T | 211 | K15803 | GERD; (-)-germacrene D synthase [EC:4.2.3.75] |
| Cl_chr06_21600T | 559 | K15803 | GERD; (-)-germacrene D synthase [EC:4.2.3.75] |
| Cl_chr07_03730T | 387 | K15803 | GERD; (-)-germacrene D synthase [EC:4.2.3.75] |
| Cl_chr07_03740T | 154 | K14184 | TPS21; alpha-humulene/beta-caryophyllene synthase [EC:4.2.3.104 4.2.3.57] |
| Cl_chr07_03750T | 565 | K14184 | TPS21; alpha-humulene/beta-caryophyllene synthase [EC:4.2.3.104 4.2.3.57] |
| Cl_chr07_03760T | 224 | K14184 | TPS21; alpha-humulene/beta-caryophyllene synthase [EC:4.2.3.104 4.2.3.57] |
| Cl_chr07_03820T | 271 | K15803 | GERD; (-)-germacrene D synthase [EC:4.2.3.75] |
| Cl_chr07_03940T | 297 | K15803 | GERD; (-)-germacrene D synthase [EC:4.2.3.75] |
| Cl_chr07_03950T | 96 | K15803 | GERD; (-)-germacrene D synthase [EC:4.2.3.75] |
| Cl_chr07_27100T | 289 | K20659 | LUS; lupeol synthase [EC:5.4.99.41] |
| Cl_chr07_32800T | 185 | K15803 | GERD; (-)-germacrene D synthase [EC:4.2.3.75] |
| Cl_chr07_32810T | 369 | K15803 | GERD; (-)-germacrene D synthase [EC:4.2.3.75] |
| Cl_chr07_32820T | 107 | K15803 | GERD; (-)-germacrene D synthase [EC:4.2.3.75] |
| Cl_chr07_32830T | 80 | K15803 | GERD; (-)-germacrene D synthase [EC:4.2.3.75] |
| Cl_chr07_32840T | 152 | K15803 | GERD; (-)-germacrene D synthase [EC:4.2.3.75] |
| Cl_chr07_32870T | 377 | K15803 | GERD; (-)-germacrene D synthase [EC:4.2.3.75] |
| Cl_chr07_32890T | 392 | K14184 | TPS21; alpha-humulene/beta-caryophyllene synthase [EC:4.2.3.104 4.2.3.57] |
| Cl_chr07_39810T | 80 | K15803 | GERD; (-)-germacrene D synthase [EC:4.2.3.75] |
| Cl_chr07_40340T | 140 | K20561 | CYP71BL2; costunolide synthase [EC:1.14.14.150] |
| Cl_chr08_03870T | 311 | K14173 | AFS1; alpha-farnesene synthase [EC:4.2.3.46] |
| Cl_chr08_10050T | 936 | K20659 | LUS; lupeol synthase [EC:5.4.99.41] |
| Cl_chr08_10070T | 101 | K20659 | LUS; lupeol synthase [EC:5.4.99.41] |
| Cl_chr08_16100T | 68 | K20659 | LUS; lupeol synthase [EC:5.4.99.41] |
| Cl_chr08_16110T | 643 | K20659 | LUS; lupeol synthase [EC:5.4.99.41] |
| Cl_chr08_19120T | 108 | K20659 | LUS; lupeol synthase [EC:5.4.99.41] |
| Cl_chr08_19140T | 140 | K20659 | LUS; lupeol synthase [EC:5.4.99.41] |
| Cl_chr08_19150T | 249 | K20659 | LUS; lupeol synthase [EC:5.4.99.41] |
| Cl_chr08_19160T | 264 | K20659 | LUS; lupeol synthase [EC:5.4.99.41] |
| Cl_chr08_23330T | 354 | K20659 | LUS; lupeol synthase [EC:5.4.99.41] |
| Cl_chr08_23340T | 280 | K20659 | LUS; lupeol synthase [EC:5.4.99.41] |
| Cl_chr08_23350T | 766 | K20659 | LUS; lupeol synthase [EC:5.4.99.41] |
| Cl_chr08_23360T | 643 | K20659 | LUS; lupeol synthase [EC:5.4.99.41] |
| Cl_chr08_23450T | 759 | K20659 | LUS; lupeol synthase [EC:5.4.99.41] |

KO, Entry no. of KEGG Orthology;
